# The prevalence of probable overactive bladder and associated risk factors among medical students in Jordan: a cross-sectional study

**DOI:** 10.1186/s12894-023-01394-4

**Published:** 2024-01-03

**Authors:** Saleh Abuorouq, Mohammad Al-Zubi, Abdullah M. Al-Ali, Laith H. Aloqaily, Malek A. Talafha, Azmi M. Migdadi, Hashem Abu Serhan

**Affiliations:** 1 Department of Clinical Medical Sciences, Urology Division, Faculty of Medicine, Yarmouk University, Irbid, Jordan; 2 Faculty of Medicine, Yarmouk University, Irbid, Jordan; 3 Department of Ophthalmology, Hamad Medical Corporations, Doha, Qatar

**Keywords:** Overactive bladder, Urgency, Urology, Medical students, Jordan

## Abstract

**Background:**

To discuss the impact of overactive bladder (OAB) on medical students. overactive bladder. is a chronic condition that causes sudden and intense urges to urinate, which can have significant physical and psychological effects on patients’ lives. The prevalence of OAB among medical students is relatively high, with some studies reporting rates as high as 35.4%. This research aims to shed light on the prevalence rates and risk factors associated with OAB among medical students in Jordan.

**Methods:**

A cross-sectional study was conducted using an online self-reported questionnaire as the study tool. The questionnaire collected the sociodemographic, health, and academic characteristics of medical students, as well as the new 7-item OABSS score.

**Results:**

Out of the total sample of medical students surveyed (n = 525), 44.5% reported experiencing symptoms of OAB. Furthermore, the analysis also revealed that there was a significant difference in the prevalence of OAB between the ages of medical students. In addition, the study also found that there was a significant association between OAB symptoms and basic years, positive history of diagnostic UTI, positive history of recent trauma, high stress, and taking certain medications.

**Conclusions:**

The study highlights the need for further research in this area and emphasizes the possible implications of OAB for medical students, including the need for additional support and resources to manage the condition.

## Background

Overactive bladder (OAB) as defined by the International Continence Society (ICS) is episodes of an intense, sudden desire to urinate right away (urgency), which is typically accompanied by frequency and nocturia with or without urge urine incontinence (UUI), if no evident pathology, such as a urinary tract infection (UTI), is present [1]. This frequent and chronic ailment can have a significant influence on patients’ lives by humiliating them, affecting their ability to function and maintain relationships, and in some circumstances forcing them to arrange their life around managing their symptoms. On a bodily level, OAB is obviously annoying or crippling, but there is evidence that the symptoms are also damaging to psychological health. Accordingly, OAB has been linked to symptoms of anxiety and depression and has been found to have a detrimental impact on health-related quality of life (QoL) [2]. Overactive bladder patients’ anxiety and depression symptoms and their OAB symptoms are both significantly connected with one another [3].

A study called EPIC was Conducted in five countries, including Canada, Germany, Italy, Sweden, and the UK showed an overall OAB prevalence of 11.8% [4]. Whereas the National Overactive Bladder Evaluation (NOBLE) program that was developed in the United States showed an overall OAB prevalence of 16% [5]. Both were conducted among adults > 18 years old with similar rates in men and women [4, 5]. Considering health college students some studies estimate the prevalence of overactive bladder, for example, a previous study showed that 21.7% of female Health Professions students at universities in the Pacific Northwest had OAB [6]. Overactive bladder symptoms were also prevalent among medical and dentistry students across Palestinian universities and a study showed that 35.4% of Turkish midwifery students have probable overactive bladder [7, 8].

Common medical disease like hypertension, diabetes mellitus and hyperlipidemia, operation history, smoking and overweight (body mass index [BMI] > = 25) are considered as significant risk factors of overactive bladder [9–11]. According to cohort study the overall incidence of psychiatric disorders in the OAB exposed group was 41.7% higher than in the non-OAB unexposed group [12]. Psychiatric disorders like stress is common among medical students while they are training [13, 14]. There are several specific stressors associated with medical school, such as exposure to pain and death in people, a highly competitive setting, ethical dilemmas, overwhelming amounts of material to learn in short periods of time, unclear expectations, and financial stressors [15, 16]. In the course of medical education, stress and poor academic performance can form a vicious circle (growing stress results in declining performance, which, in turn, raises stress) [15]. Overactive bladder is a common medical condition affecting millions of people. In the landscape of existing research on OAB, this study stands out for its distinctive focus on a previously underexplored demographic – medical students. While numerous studies have delved into the prevalence and impact of OAB among various populations, including adults and specific professional groups, the unique context of medical education remains largely uncharted. Our investigation seeks to bridge this knowledge gap by providing a comprehensive analysis of OAB within the medical student community. The novelty of our study lies in its specific emphasis on the challenges and implications of OAB in a cohort undergoing medical training, a demographic with distinct stressors and lifestyle factors that could potentially exacerbate the impact of this condition. By unraveling the prevalence rates, associated risk factors, and potential repercussions of OAB in the realm of medical education, we aim to contribute valuable insights that extend beyond the general population, thereby warranting attention and further exploration in the academic and clinical spheres.

## Materials and methods

### Study design

This cross-sectional study was conducted among medical students in Jordan using an online self-reported questionnaire as the study tool. The survey was conducted under the IRB approval at Yarmouk University and in accordance with the principles of the Helsinki Declaration Written informed consent was not obtained because this survey was performed without collecting private information such as participants’ names. Yarmouk University’s ethical committee waived the requirement for consent. The questionnaire was created with google forms survey as a study tool in English language and was distributed through email to random samples of Jordanian medical students. The study population included Jordanian medical students. Only medical students on study benches were included in the study. We calculated the sample size required for this investigation using an online sample size calculator. The sample size was calculated using a 95% confidence interval (CI) and a 5% margin of error, assuming a population of about 19,000 medical students in the different Jordanian universities, [17]. This study required 377 students as a sample size. Medical students from Jordan’s five major universities were invited to participate in the study via established email lists. The data collection was completed after 525 replies were received using a convenience sampling technique.

### Study variables

The questionnaire consists of two parts; The first part collected sociodemographic, health, and academic characteristics of the medical students like age, gender, height, body weight, smoking habits, amount of daily water intake, employment status, presence of chronic disease, history of surgery, history of trauma, history of diagnosed UTI, drug history, history of urodynamic study, academic discipline, academic stage (basic or clinical), self-rated satisfaction with the academic achievement, self-rated degree of stress (subjectively on a Likert scale, ranging from low to high), self-rated satisfaction with the financial status and self-rated satisfaction with the social life. The second part was the new 7-items Over active bladder symptom score (OABSS) which is a self-administered symptom assessment tool designed to quantify the OAB symptoms into a single score system [18]. The study was not intended to diagnose patients with OAB as it was not clinically based. However, the Overactive Bladder Symptom Score is a valid tool for assessing all aspects of Overactive Bladder [18].

The OABSS score contains 7 items; Nocturia, frequency, reason for urination, urgency duration, effect on daily activities, urge and incontinence [18]. The wording of questions was determined after consulting the urologists at the Faculty of Medicine - Yarmouk University. Each question of these seven can achieve one of 5 scores from 0 to 4, the lowest total score of the scale is 0 and the highest score is 28 after the summation of the of scores of all questions. Students with current dysuria were excluded from the study. Significant OAB was defined by a score of 8 or more [19]. Significant OAB results which score > = 1 of urge incontinence question were considered to have urinary incontinence (OAB-wet) otherwise (OAB-dry).

### Study analysis

The information was taken from Google Forms and converted to an Excel spreadsheet before being entered into the Statistical Package for Social Sciences (SPSS) version 27 (IBM SPSS Corp, SPSS Statistics ver. 26, USA). Descriptive analysis was used to display categorical variables as percentages and frequencies while presenting numerical variables as a mean and standard deviation to evaluate the data quantitatively. The significance of the data was determined using a categorical Chi-square test of independence. All statistical tests were conducted with a 95% confidence interval and a 5% error margin. A *p*-value of less than 0.05 was considered statistically significant. Tests of normality using Shapiro-Wilk test was performed showed that the data is not normally distributed (p < 0.001). A Kendall tau-b (τb) correlation was conducted to assess the relationship between statistically significant risk factors and overactive bladder diagnosis. Correlation is significant at the 0.05 level (2-tailed).

## Results

A total of 525 participants with valid responses were involved in the final analysis. The majority of participants were female (58.9%, n = 309). And those with basic years were slightly more than clinical years (53.1%, n = 279). Moreover, about one third of the participants had a BMI above 25 kg/m2. In addition, 48.4% of the students (n = 254) were not satisfied with their academic achievement. Almost 67.4% of the medical students (n = 354) were having high stress in their life. The Sociodemographic, health, and academic of the study population are shown in Table 1.

**Table 1:** Sociodemographic, health, and academic characteristics of the students who participated in the study (n = 525)

Variable	Frequency (n)	Percent (%)
Age (years)		
< 21	207	39.4
≥ 21	318	60.6
Gender		
Male	216	41.1
Female	309	58.9
Academic level*		
Basic years	279	53.1
Clinical years	246	46.9
Employment status		
Yes	12	2.3
No	513	97.7
Body mass index		
< 25	328	62.5
≥ 25	197	37.5
Presence of chronic diseases^¥^		
Yes	43	8.2
No	482	91.8
History of diagnostic urinary tract infections		
Yes	92	17.5
No	433	82.5
History of surgery		
Yes	78	14.9
No	447	85.1
History of recent trauma		
Yes	50	9.5
No	475	90.5
Smoking^€^		
Yes	99	18.9
No	426	81.1
Self-rated satisfaction with academic achievement		
Satisfied	271	51.6
Not satisfied	254	48.4
Self-rated stress		
Low stress	171	32.6
High stress	354	67.4
Self-rated satisfaction with the financial status		
Satisfied	334	63.6
Not satisfied	191	36.4
Self-rated satisfaction with the social life		
Satisfied	327	62.3
Not satisfied	198	37.7
Amount of daily water intake		
< 1.5 L	311	59.2
≥ 1.5 L	214	40.8

*Basic years include 1st -3rd years, clinical years include 4th -6th years; ¥Chronic diseases include hypertension, diabetes mellitus, immunologic diseases, and others; €Smoking includes electronic cigarettes, waterpipe, or ordinary cigarettes

Based on the cross-sectional data analysis conducted in SPSS, it was found that a significant percentage of medical students suffer from OAB symptoms. Out of the total sample of medical students surveyed (n = 525), 233.6 (44.5%) of the included students reported experiencing symptoms of OAB. Furthermore, the analysis revealed that there was a significant difference in the prevalence of OAB between ages of medical students. Specifically, 55.6% (n = 115) of medical students younger than 21 years reported experiencing OAB symptoms compared with 37.4% (n = 119) of students older than 21 years. In addition, the analysis showed that there was a significant association between OAB symptoms and being in the basic years (53.4%, n = 149), a positive history of diagnostic urinary tract infection (UTI) (58.7%, n = 54), a positive history of recent trauma (58%, n = 29), high stress (48.9%, n = 173), and taking medications such as alpha-blockers, sedative-hypnotics, anti-depressants, anti-psychotics, angiotensin-converting enzyme (ACE) inhibitors, loop diuretics or nonsteroidal anti-inflammatory drugs (60.7%, n = 37) as shown in Table 2.

**Table 2:** Risk factors of overactive bladder and their possible association

Variable	Frequency (%)	Presence of OAB* (%)	Absence of OAB (%)	*P*-value
Age (years)				**< 0.001**
< 21	207 (39.4)	115 (55.6)	92 (44.4)	
≥ 21	318 (60.6)	119 (37.4)	199 (62.6)	
Gender				0.347
Male	216 (41.1)	91 (42.1)	125 (57.9)	
Female	309 (58.9)	143 (46.3)	166 (53.7)	
Academic level				**< 0.001**
Basic years	279 (53.1)	149 (53.4)	130 (46.6)	
Clinical years	246 (46.9)	85 (34.6)	161 (65.4)	
Employment status				0.838
Yes	12 (2.3)	5 (41.7)	7 (58.3%)	
No	513 (97.7)	229 (44.6)	284 (55.4)	
Body mass index				0.691
< 25	328 (62.5)	144 (43.9)	184 (56.1)	
≥ 25	197 (37.5)	90 (45.7)	107 (54.3)	
Presence of chronic diseases				0.220
Yes	43 (8.2)	23 (53.5)	20 (46.5)	
No	482 (91.8)	211 (43.8)	271 (56.2)	
History of diagnostic urinary tract infections				**0.003**
Yes	92 (17.5)	54 (58.7)	38 (41.3)	
No	433 (82.5)	180 (41.6)	253 (58.4)	
History of surgery				0.296
Yes	78 (14.9)	39 (50)	39 (50)	
No	447 (85.1)	195 (43.6)	252 (56.4)	
History of recent trauma				**0.045**
Yes	50 (9.5)	29 (58)	21 (42)	
No	475 (90.5)	205 (43.2)	270 (56.8)	
Smoking				0.633
Yes	99 (18.9)	42 (42.4)	57 (57.6)	
No	426 (81.1)	192 (45.1)	234 (54.9)	
Self-rated satisfaction with academic achievement				0.233
Satisfied	271 (51.6)	114 (42.1)	157 (57.9)	
Not satisfied	254 (48.4)	120 (47.2)	134 (52.8)	
Self-rated stress				**0.004**
Low stress	171 (32.6)	61 (35.7)	110 (64.3)	
High stress	354 (67.4)	173 (48.9)	181 (51.1)	
Self-rated satisfaction with the financial status				0.106
Satisfied	334 (63.6)	140 (41.9)	194 (58.1)	
Not satisfied	191 (36.4)	94 (49.2)	97 (50.8)	
Self-rated satisfaction with the social life				0.497
Satisfied	327 (62.3)	142 (43.4)	185 (56.6)	
Not satisfied	198 (37.7)	92 (46.5)	106 (53.5)	
Amount of daily water intake				0.254
< 1.5 L	311 (59.2)	145 (46.6)	166 (53.4)	
≥ 1.5 L	214 (40.8)	89 (41.6)	125 (58.4)	
Taking medications^¥^				**0.007**
Yes	61 (11.6)	37 (60.7)	24 (39.3)	
No	464 (88.4)	197 (42.5)	267 (57.5)	
Urodynamic study				0.317
Normal	26 (5)	10 (38.5)	16 (61.5)	
Overactive	5 (1)	4 (80)	1 (20)	
Hypotonic	3 (0.6)	2 (66.7)	1 (33.3)	
Not performed	491 (93.5)	218 (44.4)	273 (55.6)	

*Overactive bladder (OAB) diagnosis was made if the total score is ≥ 8; ¥Medications include alpha-blockers, sedative-hypnotics, anti-depressants, anti-psychotics, ACE inhibitors, loop diuretics, nonsteroidal anti-inflammatory drugs; *P*-value in bold is statistically significant

Using correlation coefficient (τb) between overactive bladder and significant associated risk factors, we found that age, academic level, presence of history of diagnostic UTI, presence of history of recent trauma, self-rated stress and taking medications like alpha-blockers, sedative-hypnotics, anti-depressants, anti-psychotics, ACE inhibitors, loop diuretics, nonsteroidal anti-inflammatory drugs is strongly correlated to the severity of OAB diagnosis (Table 3). Table 4 summarized the key features of our study.

**Table 3:** Correlation Coefficient (τb) between overactive bladder and significant associated risk factors

Variable	Τb	N	Sig
Age	−0.189	525	< 0.001
Academic level	−0.178	525	< 0.001
History of diagnostic urinary tract infections	−0.131	525	0.003
History of recent trauma	−0.088	525	0.045
Self-rated stress	0.124	525	0.004
Taking medications	0.117	525	0.007

**Table 4:** Summary of the key features of our study

Characteristic	Percentage/Frequency
Total Participants	525
Female	58.9% (n = 309)
Basic Academic Level	53.1% (n = 279)
BMI > 25 kg/m^2	33.3%
Dissatisfaction with Academics	48.4% (n = 254)
High Stress	67.4% (n = 354)
OAB Symptoms	**Percentage/Frequency**
Total with OAB Symptoms	44.5% (n = 233)
Age < 21 years	55.6% (n = 115)
Basic Academic Level	53.4% (n = 149)
History of Diagnostic UTI	58.7% (n = 54)
History of Recent Trauma	58.0% (n = 29)
High Stress	48.9% (n = 173)
Medications (Alpha-blockers, etc.)	60.7% (n = 37)
Correlation Coefficients (τb)	
Age	Strongly correlated
Academic Level	Strongly correlated
History of Diagnostic UTI	Strongly correlated
Recent Trauma	Strongly correlated
Self-rated Stress	Strongly correlated
Medications (Alpha-blockers, etc.)	Strongly correlated

## Discussion

Epidemiological studies are essential to delineate the medical and social aspects of a disease. Although OAB is highly prevalent in the population, it’s difficult to estimate the accurate percentage of it. This is attributed to many factors, among the most important ones is that there is no tool for diagnosing OAB; the majority of cases tend to be discovered by subjective symptoms rather than objective routine examination by healthcare providers; so the patient statements may be sufficient for the diagnosis. This self-perception period of symptoms could result in a delay to visit a healthcare provider. To our knowledge, this is the first cross-sectional study which uses the OABSS to estimate the prevalence of OAB among medical students in Jordan and the associated sociodemographic, health and academic factors trying to control the modifiable ones to reduce the future prevalence.

In our study, 42.1% of males and 46.3% of females (overall 44.57%) met the criteria for OAB (OABSS). When these findings are compared to the prevalence of OAB in Milsom et al. (16% of men, 17% of women (overall 16.6%)) and a US population-based study (16.9% of women, 17% of men (overall 16.5%)) a significant difference is observed ; this may be attributable to the fact that OAB is generally affected by a variety of factors such as age, gender, chronic diseases, geographical location and population type; medical students are confronted with more stressful events during the course of their studies than the general population [20].

The definition of OAB used appears to be a major factor. In the European study, OAB was defined as any symptoms involving frequency (more than eight voids/day), nocturia (two or more voids/ night), urgency, and urgency incontinence of any frequency or severity [20]. In the USA study, OAB was defined by urgency (four or more times/month) and either frequency (more than eight voids/day) or use of any coping methods [5]. To be classed as having OAB with incontinence, a respondent with OAB had to have three or more incidents each month. We classified OAB in this study as eight or more voids per day and one or more urgency episodes per week. OAB with incontinence was defined as one or more episode/week in addition to voiding frequency and urgency.

Furthermore, our study showed a significant association between OAB and age, academic level, history of UTI, history of recent trauma, medications use with *p*-values of (< 0.001, < 0.001, 0.003, 0.045, 0.007) respectively.

Age-related changes in the bladder and pelvic floor tissues and/or in the nervous system contribute to the high prevalence of OAB in elderly. Increased incidence of OAB with age may be linked to cerebrovascular disorders and pelvic tumors [21]. In contrast to previous studies of OAB, which found that the prevalence of OAB increases with age, our study found a higher percentage of OAB in medical students younger than 21 years old compared to those older than 21 years old (OR = 2.1); this may be attributed to the fact that our sample was drawn from a young-aged population with a negligible age difference (17–24 year-old0) [5, 20, 22].Our study has also revealed that OAB was substantially more prevalent in basic years medical students than in clinical years (OR = 2.165). In contrast, a prior cross-sectional study among Palestinian medical students found no significant difference between the two groups (*p* value = 0.329).

The deviation from traditional age-related trends in OAB incidence prompts a multifaceted exploration. Most importantly, stress, inherent to the demanding academic environment of basic medical education, emerges as a significant factor [23]. The psychosocial stressors, coupled with lifestyle influences and physiological considerations unique to younger individuals, may collectively contribute to the observed prevalence of OAB [24]. This demographic’s heightened susceptibility to stress-induced dysregulation of the nervous system may further amplify OAB symptoms.

Bladder overactivity and urinary incontinence may be exacerbated by stress, anxiety, and depression may actually contribute to. Although the specific explanation is unknown, there are some possibilities. ; the first is that stress creates the so-called fight-or-flight response which enhances the sensitivity of the nervous system [23]. The second argument is that anxiety and stress can lead to muscle tension, which affects the bladder muscles and increase the urge to urinate [23].

Our study found an association between stress experienced by medical students during the course of their studies and the prevalence of OAB among them (*P*-value = 0.004). Students who reported a stressful life had higher OAB symptoms than those who reported a less stressful life (OR = 1.72). This is in accordance with the findings of numerous prior studies [24].

The intricate relationship between psychological stress and overactive bladder (OAB) symptoms unfolds through a complex interplay of stress-induced hormonal responses [25]. Chronic stress unleashes cortisol and catecholamines, key stress hormones, initiating a cascade of physiological changes. Elevated cortisol levels, emblematic of chronic stress, activate the sympathetic nervous system, inducing detrusor muscle relaxation during bladder filling phase, contributing to overfilling followed by urgency and frequency—the hallmark symptoms of OAB [26]. Simultaneously, catecholamines directly impact detrusor muscle activity, intensifying the sensation of urgency [26]. Stress-induced neurotransmitter modulation and the release of pro-inflammatory mediators further contribute to altered bladder sensitivity and detrusor overactivity [27].

Subjects with ‘’urinary infection or other apparent pathology’’ should be excluded from the diagnosis of overactive bladder, according to the standardization report [28]. Identification of overactive bladder without ruling out recognized causes of urgency may result in an overestimation of prevalence. Subjects with a recent urinary tract infection were eliminated. Kajiwara et al. discovered a link between OAB in children and a history of UTI and nocturnal enuresis [29]. According to a Chinese survey, children with a history of UTI and nocturnal enuresis had a higher prevalence of OAB than normal children [30]. Similarly, our study found a link between a history of UTI and OAB (*p* value = 0.003). Although the link between a history of UTI and OAB is uncertain. UTI, particularly cystitis, is well recognized to produce involuntary detrusor overactivity [20]. Although UTIs are treatable, symptoms of detrusor overactivity can remain and impair bladder function.Except for a prior study conducted among medical students in Palestine, a history of recent trauma has not previously been associated to the development of OAB symptoms. The study discovered no link between a history of recent trauma and OAB symptoms (*P*-value = 0.328) [7]. In our study, OAB symptoms were shown to be more common in medical students with a history of recent trauma (OR = 1.82%). We believe that trauma can be detrimental in ways that are educational and psychological in addition to physical.

Drugs are frequently metabolized and excreted in the urine, the lower urinary tract is particularly vulnerable to adverse effects. Furthermore, when drug metabolites are retained in the bladder, they are in close contact to the epithelium for extended periods of time. Stress incontinence, urge incontinence, or overflow incontinence may result from the medications [31]. Several drugs, including alpha1-adrenoceptor antagonists, antipsychotics, and antidepressants, have been identified as potential causes of drug-induced urine incontinence [32]. So far as we know, this is the first study to look for a direct link between particular drugs and OAB. OAB symptoms were shown to be more common among medical students who took alpha-blockers, sedative-hypnotics, antidepressants, antipsychotics, ACE inhibitors, loop diuretics, and nonsteroidal anti-inflammatory medicines (OR = 2.1). In terms of gender, our study found that both genders had virtually comparable prevalence of OAB symptoms (*P*-value = 0.347). Milsom et al. [20] were similar. However, the bulk of research have found that females have a higher prevalence of overactive bladder [7]. Our study cohort included medical students in their twenties, with the majority of them being medically free; this could explain our finding that there is an insignificant connection between OAB and the occurrence of chronic disorders (*P*-value = 0.220). In contrast to other studies [7]. Occupational stress exposure is one of the important factors which may raise the prevalence of OAB, by this way occupation may have considerable impact on the prevalence of OAB, and that what was found in the literature [7, 22]. Unlikely, our study found that there’s no significant link between OAB and employment status (*P*-value = 0.838); which could be attributable to the fact that most of medical students included in our study are unemployed.

Like the Kim et al. study ,our study indicated that smoking had insignificant effect on OAB prevalence among medical students in Jordan (*P*-value = 0.633) [22]. The reason for the association between obesity and OAB is not clear, though it has been claimed that obesity might may produce structural changes in the body, resulting in pelvic organ compression and adverse effects regarding bladder function [33]. The link between high BMI and OAB was insignificant (*P*-value = 0.691). This was comparable to C. Teloken et al. findings [32], However, many other studies had shown significant relationship between obesity and OAB [22].

The nexus between obesity and overactive bladder (OAB) is extended to encompass systemic inflammation, a critical aspect often associated with obesity and implicated in the manifestation of OAB symptoms [27]. Obesity induces chronic low-grade inflammation characterized by elevated pro-inflammatory cytokines [34]. This inflammatory milieu, reaching the lower urinary tract, contributes to detrusor overactivity and heightened bladder sensitivity, characteristic of OAB. Adipose tissue, functioning as an endocrine organ, releases adipokines, with imbalances further fueling the inflammatory environment [34]. Notably, leptin, an elevated adipokine in obesity, may play a role in neurogenic inflammation affecting bladder function [35]. Additionally, obesity-associated insulin resistance intertwines with inflammatory pathways, potentially exacerbating OAB symptoms [36].

A study by Kraus et al. [37] aimed to assess costs, patient encounters, adherence, and persistence in OAB patients starting mirabegron (MIRA) and antimuscarinic (AM) combination therapy. Their results showed that monthly all-cause healthcare costs were generally similar across groups, except for those transitioning to OAB procedures, which incurred higher costs. Treatment persistence on combination therapy was comparable to MIRA monotherapy and potentially higher than AM monotherapy, while adherence through day 365 was 26%.

Another interesting study by Chermansky et al. [38]. indicated that various factors associated with poor outcomes for Botulinum Toxin-A (BTX-A), including increasing age, male sex, and frailty. Sacral neuromodulation (SNM) outcomes were influenced by factors such as psychiatric comorbidity and lead placement procedures. Also, Percutaneous Tibial Nerve Stimulation (PTNS) success was associated with increased daytime frequency and lower first sensation of bladder filling.

A paper by Marand et al. [39] suggests a potential link between the urinary microbiome and a specific phenotype of OAB, indicating a new avenue for studying the causes and treatments of OAB.

Some limitations were encountered in this survey. First, in conducting this study, we recognize the inherent limitation of relying on a self-reported questionnaire for the assessment of overactive bladder (OAB) symptoms and the fact that participants didn’t underwent a healthcare investigation which may have resulted in information bias. Furthermore, asymptomatic UTI patients and those who are unaware of UTI symptoms were not excluded. Second, the design is cross-sectional so causal relationships cannot be documented. Third, studies conducted among medical students regarding OAB are few. Moreover, while we acknowledge the importance of excluding various pathologies beyond UTIs, such as hematuria, as the choice of dysuria as an exclusion criterion was made with the intention of focusing on symptoms directly associated with OAB, and it was selected based on its clinical relevance to urgency and frequency, key components of OAB as defined by the International Continence Society.

## Conclusion

In conclusion, this research highlights a high prevalence of overactive bladder symptoms (OABSS) among medical students, with 44.5% reporting such symptoms. The study findings suggest that OABSS was more common among younger students and those in their early years of medical education. The presence of risk factors such as a recent history of UTI, trauma, and high stress was significantly associated with the development of OAB symptoms. These findings have implications for the management of OAB symptoms among medical students, including targeted interventions to reduce stress and improve mental health, as well as the need for greater awareness of the risk factors for OAB among this population. Overall, this study emphasizes the need of diagnosing and treating OAB symptoms in medical students in order to support their success.

## Data Availability

All data generated or analyzed during this study are included in this published article.

## Abbreviations

OAB: Overactive bladder
ICS: International Continence Society
UUI: Urge urine incontinence
UTI: Urinary tract infection
QoL: Quality of life
NOBLE: National Overactive Bladder Evaluation
BMI: Body mass index
OABASS: Over active bladder symptom score
